# GRIEVOUS: your command-line general for resolving cross-dataset genotype inconsistencies

**DOI:** 10.1093/bioinformatics/btae489

**Published:** 2024-07-30

**Authors:** James V Talwar, Adam Klie, Meghana S Pagadala, Hannah Carter

**Affiliations:** Division of Medical Genetics, Department of Medicine, University of California San Diego, La Jolla, CA 92093, United States; Bioinformatics and Systems Biology Program, University of California San Diego, La Jolla, CA 92093, United States; Division of Medical Genetics, Department of Medicine, University of California San Diego, La Jolla, CA 92093, United States; Bioinformatics and Systems Biology Program, University of California San Diego, La Jolla, CA 92093, United States; Biomedical Science Program, University of California San Diego, La Jolla, CA 92093, United States; Division of Medical Genetics, Department of Medicine, University of California San Diego, La Jolla, CA 92093, United States; Bioinformatics and Systems Biology Program, University of California San Diego, La Jolla, CA 92093, United States; Moores Cancer Center, University of California San Diego, La Jolla, CA 92093, United States

## Abstract

**Summary:**

Harmonizing variant indexing and allele assignments across datasets is crucial for data integrity in cross-dataset studies such as multi-cohort genome-wide association studies, meta-analyses, and the development, validation, and application of polygenic risk scores. Ensuring this indexing and allele consistency is a laborious, time-consuming, and error-prone process requiring a certain degree of computational proficiency. Here, we introduce GRIEVOUS, a command-line tool for cross-dataset variant homogenization. By means of an internal database and a custom indexing methodology, GRIEVOUS identifies, formats, and aligns all biallelic single nucleotide polymorphisms (SNPs) across all summary statistic and genotype files of interest. Upon completion of dataset harmonization, GRIEVOUS can also be used to extract the maximal set of biallelic SNPs common to all datasets.

**Availability and implementation:**

GRIEVOUS and all supporting documentation and tutorials can be found at https://github.com/jvtalwar/GRIEVOUS. It is freely and publicly available under the MIT license and can be installed via pip.

## 1 Introduction

The explosion of genetic variant datasets, driven by increasingly affordable genomic profiling technologies, presents exciting opportunities across a number of fields, ranging from precision medicine to ecology to agriculture. However, taking full advantage of genetic data from multiple sources can be challenging when datasets use different conventions for encoding variants. Analyses requiring the integration of various datasets range from the straightforward, such as validating genetic findings or polygenic scores in new datasets, to the more complex, such as cross-dataset joint statistical analyses [e.g. genome wide association meta-analyses (Zeggini and Ioannidis 2009, Evangelou and Ioannidis 2013), two-sample Mendelian randomizations (Hartwig *et al.* 2016, Sanderson *et al.* 2022)]. These approaches can frequently be undermined by discrepancies in the definition of identical variants across datasets (Zeggini and Ioannidis 2009, Hartwig *et al.* 2016).

Common variants, such as single nucleotide polymorphisms (SNPs), are abundantly shared across different datasets, and can be imputed against reference panels to increase coverage (van Leeuwen *et al.* 2015, Coleman *et al.* 2016). Quality control of SNP data is critical, and best practices include setting uncertain genotypes to missing, orienting genotypes to the forward reference DNA strand, and filtering on missingness, minor allele frequency, or deviation from Hardy Weinberg equilibrium (Anderson *et al.* 2010, Coleman *et al.* 2016, Marees *et al.* 2018, Choi *et al.* 2020). However, even after performing the steps to generate a high-quality dataset, there can still be discrepancies between datasets due to differences in conventions used to define genotypes. If left uncorrected, these discrepancies can create illusory genotypic differences where none exist. Given that common variant sets can include millions of SNPs, it can be challenging and time consuming to manually identify and resolve discrepancies.

To reduce this burden to the researcher, we developed a **G**eneralized **R**ealignment of **I**nnocuous and **E**ssential **V**ariants **O**therwise **U**tilized as **S**kewed or GRIEVOUS, a command-line tool designed to ensure cross-cohort consistency and maximal feature recovery of biallelic SNPs, the most commonly used class of variants for genetic studies (Choi *et al.*2020). Whether creating a composite cohort from smaller studies for joint analyses, or ensuring feature fidelity for validation studies or polygenic score portability, GRIEVOUS reduces the problem of variant consistency and recovery to a simple streamlined set of commands.

## 2 Materials and methods

### 2.1 Datasets

Summary statistics and genotype data were obtained for two diseases: breast cancer (BC) and prostate cancer (PC). Specifically, data consisted of one summary statistic file and two different genotype datasets, each of which diverged in genotyping and imputation methodology from the other. Each file (i.e. both summary statistics and genotypes), for each condition studied here, indexed variants in a unique manner, capturing the discordance that can exist between datasets.

For BC, summary statistics were obtained from a large-scale BC GWAS study by Michailidou *et al.* (2017) (downloaded from: https://bcac.ccge.medschl.cam.ac.uk/bcacdata/oncoarray/oncoarray-and-combined-summary-result/gwas-summary-results-breast-cancer-risk-2017), and the genotype datasets utilized were from the Discovery, Biology, and Risk of Inherited Variants in Breast Cancer (DRIVE; dbGaP study accession: phs001265.v1.p1) project and the UK Biobank (UKBB). For PC, summary statistics from a large-scale PC multi-ancestry GWAS meta-analysis by Conti *et al.* (2021) were downloaded from dbGaP (study accession: phs001120.v2.p1). Genomic data from the ELucidating Loci Involved in Prostate cancer SuscEptibility (ELLIPSE; dbGaP study accession: phs001120.v1.p1) consortium and UKBB were utilized as the PC genotype datasets. PC PRSs were calculated as an effect size weighted genotype summation, with effect sizes and *P*-values for SNP subselection derived from the Conti *et al.* (2021) summary statistics. Finally, we note that both DRIVE and ELLIPSE were subsets of each disease’s summary statistic GWAS.

Both DRIVE and ELLIPSE were genotyped using the OncoArray microarray (Amos *et al.* 2017), while the UKBB was genotyped using the UK Biobank Axiom Array (Bycroft *et al.* 2018). To recover untyped markers in both DRIVE and ELLIPSE, we imputed genotypes with Minimac4 using the 1000 Genomes Phase 3 Version 5 reference panel, via the Michigan Imputation Server (Das *et al.*2016). Details of UKBB imputation can be found in the original UKBB report by Bycroft *et al.* (2018)

### 2.2 GRIEVOUS: design and framework

**Figure 1.**
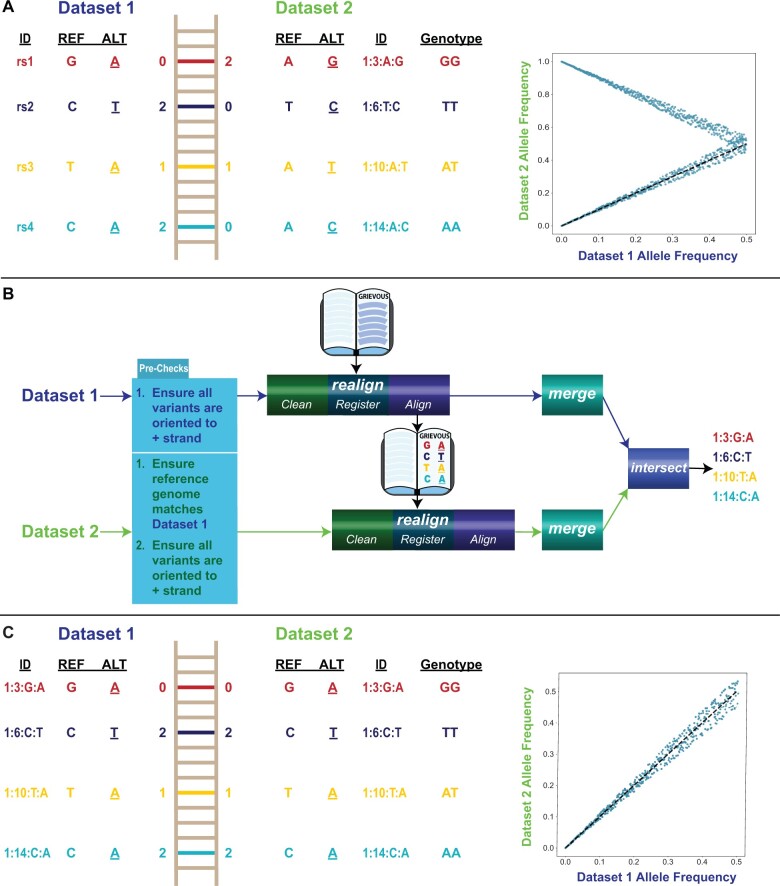
(A) Example of the same genotypes described by two different genomic datasets that use different schemas for indexing and assigning variants. This complicates variant recovery and leads to spurious artifactual allele frequency divergences across datasets. (B) Each genomic dataset is harmonized with GRIEVOUS. Upon ensuring all datasets use the same reference assembly and strand orientation, each dataset is passed through *realign*, sequentially, and then passed to *merge* to generate composite dataset level reports of all identified biallelic and inverted variants resulting from the GRIEVOUS realignment process. Finally, *intersect* is called once across all datasets, to identify the maximal set of biallelic SNPs common across all datasets. (C) After harmonization with GRIEVOUS, all genomic datasets use the same schema of variant indexing, and all biallelic SNPs common across all datasets are consistently assigned, eliminating cross-dataset artifactual allele frequency divergences.

The current state of variant indexing can be summarized concisely as unsystematic. Datasets can use any means or mechanisms for indexing variants, ranging from custom concatenations of dataset variant information (e.g. CHR_POS_REF_ALT), to rsIDs, to any mixture in between (Fig. 1A). Arbitrary assignments of reference (REF) and alternate (ALT) definitions across datasets poses a similar problem (Fig. 1A). Differing genotyping technologies, and in the case of summary statistics, the characterization of effect alleles, often lead to divergences in REF/ALT definitions across datasets. Harmonization of dataset indices and allele assignments, thus first requires variant organization, a task specifically addressed by the design of GRIEVOUS (Fig. 1B).

GRIEVOUS deploys a flexible internal database, backed by chromosome-level parquet files, to build a unified variant index and align allele assignments. Initialized in an empty manner, GRIEVOUS databases are continuously updated with each run, adding all hitherto unobserved biallelic SNPs identified in a given dataset (Fig. 1B). The iterative nature of database variant additions ensures that each subsequent dataset is consistently oriented with the set of common biallelic SNPs found in all previously GRIEVOUS realigned datasets.

The key command behind GRIEVOUS is *realign*, which consists of three steps: *clean*, *register*, and *align* (Supplementary Fig. S1). *Clean* identifies valid biallelic SNPs, by means of a graph formulation (Supplementary Methods: *Identifying Valid Variants*), and resolves duplications and multiple indexing issues. Specifically, variant positions are defined as nodes and dataset-specific IDs as edges. Any node for which there exists an edge to another node in the graph is considered an invalid variant, with a dataset-specific ID pointing to multiple genomic positions. *Register* performs variant indexing reformulation (i.e. a colon-separated unification of chromosome, position, REF, and ALT defined by the dataset; CHR:POS:REF:ALT) and database comparison. Dataset variants found in the reverse index assignment from a GRIEVOUS database are marked for realignment, while all variants not found in the database by genomic position are registered for addition. *Align* enacts the adjustment of SNP assignments, updates the database, and writes output files for the *realign* run. These files include reports of identified, reassigned, and duplicated variants, and for PLINK2 binary file inputs(Purcell *et al.* 2007, Chang *et al.*2015), reassignment files to convert the binary genomic data to the GRIEVOUS format.

GRIEVOUS can harmonize an arbitrary number of user-defined genomic datasets, whether they be PLINK2 binary files, summary statistics, or a mixture of the two (Fig. 1C). Variants are processed at the chromosome-level, allowing parallelization and integration into different workflows. Additional commands, *merge* and *intersect*, combine chromosome-level reports and generate the maximal set of biallelic SNPs common to all datasets of interest, respectively (Fig. 1B). GRIEVOUS also supports the creation of multiple databases for project-specific needs, enhancing parallel processing and organization.

## 3 Results

Polygenic risk scores (PRS) provide an example of an application where performance and portability depend on the fidelity of SNP assignments across datasets. They are traditionally calculated as an effect size weighted genotype summation, wherein each SNP’s effect size is multiplied by the number of corresponding alleles carried, and these products are summed across risk alleles. Thus, if differences exist in how genotypes are defined across datasets, the contribution of discrepant SNPs to the score could be calculated incorrectly, introducing noise to the PRS calculation and reducing score performance on the outcome of interest.

This problem can be avoided through careful harmonization of SNP consistency across datasets prior to PRS calculation. GRIEVOUS accomplishes this through indexing unification, assignment synchronization, and variant recovery across datasets. To demonstrate GRIEVOUS’ effectiveness, we applied it to harmonize summary statistics and genotype data for two different diseases: breast cancer (BC) and prostate cancer (PC; *Materials and methods: Datasets*).

For each condition, GRIEVOUS *realign* was applied sequentially in a dataset consistent order: (i) summary statistics, (ii) DRIVE/ELLIPSE genotypes, (iii) UKBB genotypes. This process aligned the ALT allele in the genotyped datasets to the effect allele in the summary statistics for all biallelic variants, facilitating unbiased cross-disease comparison of SNP reassignments. The results are presented in Table 1.

**Table 1.** Summary of datasets and GRIEVOUS realignment results.

Condition	Dataset	Total number of variants	Number of biallelic SNPs identified	Number of biallelic SNPs reassigned	Average run time (minutes)	Max memory usage (GB)	Cross-dataset SNP intersection size
Breast cancer	Michailidou *et al.*^a^	11 792 542	10 413 027	0^b^	1.71 ± 1.08	3.00	10 295 993
	DRIVE	48 838 144	44 849 194	2	9.13 ± 4.03	9.97	
	UKBB	97 013 422	92 429 914	659	8.03 ± 3.69	14.77	
Prostate cancer	Conti *et al.*^a^	29 235 255	26 529 293	0^b^	4.66 ± 3.48	6.80	26 098 297
	ELLIPSE	48 899 508	44 905 050	11 329 170	7.22 ± 3.49	8.55	
	UKBB	97 013 422	92 424 521	11 374 523	10.42 ± 7.85	14.96	

a Summary statistics.

b The first dataset GRIEVOUS realigned for a condition, which by definition will reassign 0 SNPs. All GRIEVOUS realignments were deployed in parallel and used multiple cores (summary statistics: 2; genotype files: 4).

Both ELLIPSE and DRIVE used different nomenclature for common variant indexing relative to the UKBB, a disparity that GRIEVOUS effectively managed. Post-reindexing, GRIEVOUS successfully recovered and reassigned all database-consistent biallelic SNPs across datasets. For BC, there was high concordance in variant assignment, likely because DRIVE was part of the BC GWAS. However, in PC, despite ELLIPSE being part of the PC GWAS, 43.4% of cross-dataset common variants differed in allele assignment from the summary statistics and required reassignment. These discrepancies drastically impacted PRS generalization in both PC genotype datasets. Specifically, PRSs applied to the unharmonized PC datasets exhibited an area under the receiver operating characteristic curve (AUC) below random chance. However, after harmonizing all datasets with GRIEVOUS, expected PRS performance was restored (Supplementary Table S1).

In summary, GRIEVOUS harmonized datasets and identified the shared set of biallelic SNPs for both diseases, demonstrating its utility in genomic dataset harmonization.

## 4 Conclusion

The harmonization of variant indexing and assignments is crucial for the accuracy of multi-dataset studies of genetic variants such as the development, validation, and application of PRSs. Here we introduce GRIEVOUS, a command-line tool designed to simplify and expedite the process of harmonizing and recovering variants across different cohorts. GRIEVOUS efficiently unifies variant indices, and primarily focuses on the recovery of biallelic SNPs. Variants not fitting this category currently still require manual verification by the user to ensure allele assignment consistency. However, these could be supported in future releases. Before using GRIEVOUS, it is important for users to confirm that all datasets use the same reference assembly and strand orientation to ensure accurate results.

In summary, GRIEVOUS streamlines the time-consuming and meticulous process of SNP homogenization and reduces opportunities for human error.

## Supplementary Material

btae489_Supplementary_Data

## Data Availability

In order to promote the widespread use of GRIEVOUS, we have made it freely available under the MIT license and easily installable via pip. The code for GRIEVOUS, which was implemented in Python, along with supporting documentation and tutorials, can be found on GitHub (https://github.com/jvtalwar/GRIEVOUS). The code for GRIEVOUS is also archived on Zenodo (Talwar 2024).
