# Supplementary material for: GRIEVOUS: your command-line general for resolving cross-dataset genotype inconsistencies
Source: Bioinformatics. 2024 Jul 30;40(8):btae489. doi: 10.1093/bioinformatics/btae489 (PMC11322043; doi:10.1093/bioinformatics/btae489)
Supplement: btae489_Supplementary_Data [file btae489_supplementary_data.pdf]

Supplementary Methods:

Identifying Valid Variants:

To address the problem of variant index validity (i.e., ensuring variant indices are unique by genomic position), GRIEVOUS reformulates a given genomic dataset as a graph. Each unique variant genomic position is defined as a node with its corresponding edges assigned to all dataset-defined indices for that variant position. As the set of all dataset-defined indices must be unique by genomic position for a variant to be valid, all valid variants will only contain self-edges. However, all nodes existing in a connected component with a size of 2 or more, correspond to invalid variants and are removed by GRIEVOUS, as there exists at least one non-unique dataset-defined index spanning multiple genomic positions for each variant in the component (and thus a non-self edge connecting these nodes in the graph). This procedure is implemented during GRIEVOUS *realign*-ment and is illustrated in **Supplementary Fig. S1** as *Extract Valid SNPs*.

Supplementary Figures:

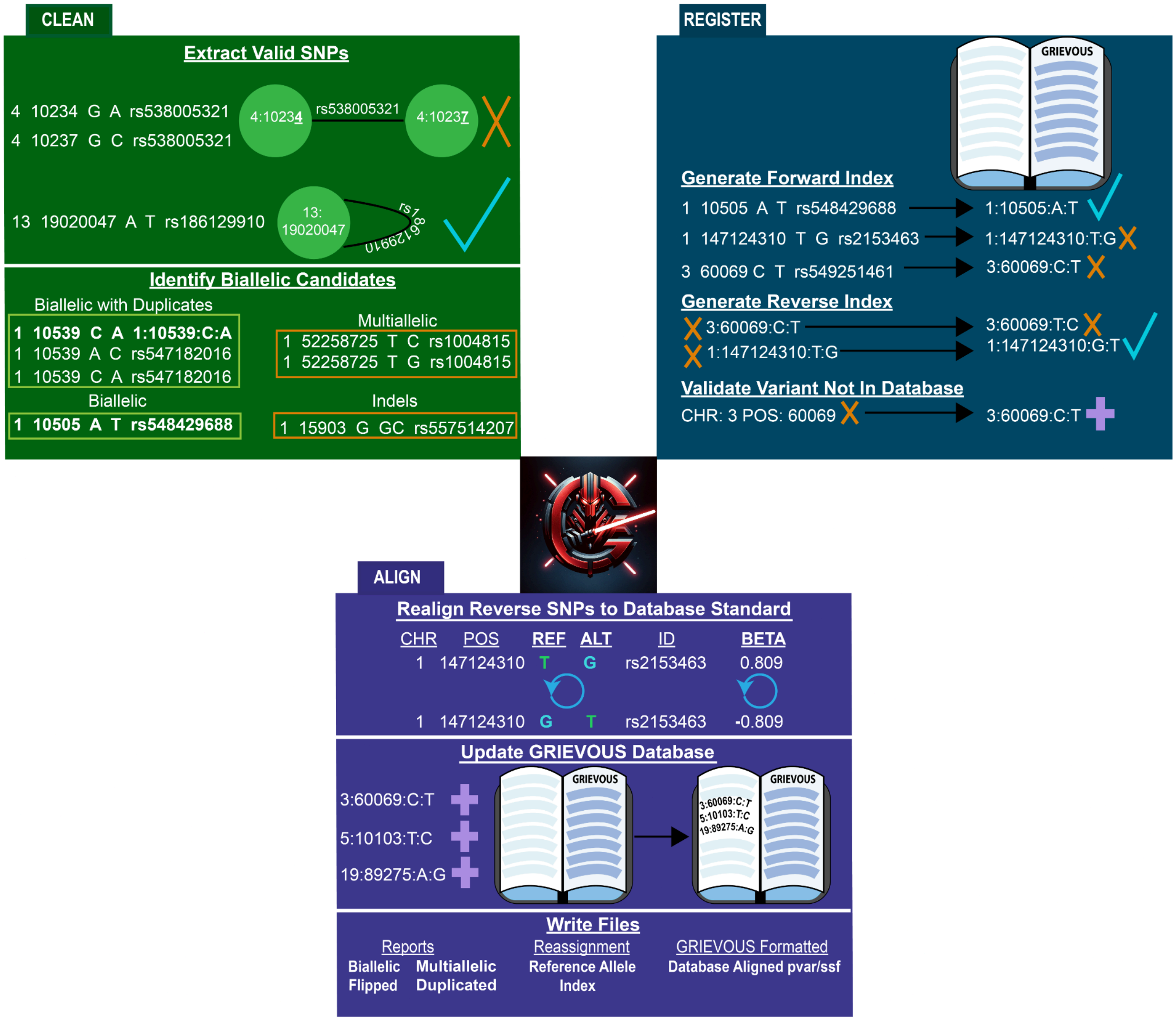

Supplementary Figure S1: Dataset realignment with GRIEVOUS can be conceptualized as a three-step workflow. The first step, *clean*, identifies and extracts valid biallelic variant candidates. The second, *register*, generates GRIEVOUS variant indices and performs variant database comparisons, identifying all biallelic variants requiring realignment and all biallelic SNP database additions. Finally, *align*, realigns REF and ALT alleles (and corresponding effect sizes for summary statistics) for those SNPs *register* identified as database reversed. Subsequently, *align* updates the GRIEVOUS database with all biallelic SNPs identified in the current dataset and writes dataset-level reports, reassignment files, and GRIEVOUS formatted files.

Supplementary Tables:

P-value Threshold	Number of PC PRS SNPs	***Before***: **ELLIPSE PRS AUC**	***After***: **ELLIPSE PRS AUC**	***Before***: **UKBB PRS AUC**	***After***: **UKBB PRS AUC**
$5x10^{-8}$	21,915	0.43673	0.63296	0.45324	0.62266
$5x10^{-7}$	28,420	0.43212	0.63744	0.44743	0.62364
$5x10^{-6}$	39,290	0.43230	0.63947	0.44056	0.62427
$5x10^{-5}$	58,326	0.43055	0.64101	0.43210	0.62585
$5x10^{-4}$	110,287	0.42048	0.64312	0.42799	0.62436

Supplementary Table S1: Polygenic risk score (PRS) area under the receiver operating characteristic curve (AUC) performances for prostate cancer (PC) genotype datasets before and after harmonization with GRIEVOUS. Current PC PRS AUCs fall in the range of 0.59-0.64[1,2], on par with PRS performances after harmonization with GRIEVOUS.

Supplementary References:

1. Helfand, B. T., Kearns, J., Conran, C. & Xu, J. Clinical validity and utility of genetic risk scores in prostate cancer. *Asian J. Androl.* **18**, 509–514 (2016).
2. Sipeky, C. *et al.* Prostate cancer risk prediction using a polygenic risk score. *Sci. Rep.* **10**, 17075 (2020).
